# Self-Evaluation in AI-Assisted Cognition: An Explanatory Framework for Calibration and Miscalibration Effects

**DOI:** 10.3390/jintelligence14070112

**Published:** 2026-06-23

**Authors:** Monica Maier

**Affiliations:** Department of Specialty with a Psycho-Pedagogical Profile, Technical University of Cluj-Napoca, 400114 Cluj-Napoca, Romania; monica.maier@dspp.utcluj.ro

**Keywords:** self-evaluation, AI-assisted cognition, judgment calibration, miscalibration, metacognitive monitoring, self-regulated learning, cognitive offloading, trust in AI

## Abstract

Generative Artificial Intelligence (AI), particularly large language models, has changed the conditions under which individuals judge their own cognitive performance. While AI-assisted tools can improve task outcomes, such improvements do not necessarily lead to more accurate self-evaluation. This article develops an integrative conceptual review of calibration and miscalibration in AI-assisted cognition. Drawing on research on metacognitive monitoring, self-regulated learning, judgment calibration, cognitive offloading, cognitive engagement, and trust in AI, the article identifies a central gap in the literature: the lack of an explanatory framework showing how AI-supported performance becomes a cue for users’ judgments of their own competence. To address this gap, the article proposes an eight-axis explanatory framework organized around the functional position of AI in the task, reflective support versus cognitive substitution, metacognitive engagement, effort redistribution, cognitive engagement, the distinction between assisted performance and actual learning, trust regulation and attribution of success, and self-evaluation accuracy. The framework is presented through qualitative relational expressions and a synthetic conceptual figure, not as an empirically estimated model. Its main contribution is to explain why AI may support calibration when it sustains reflection, verification, and learning, but may contribute to miscalibration when it promotes cognitive substitution, effort reduction, overreliance, or erroneous attribution of success. The article offers a conceptual basis for future empirical research on self-evaluation accuracy in human–AI interaction.

## 1. Introduction

The rapid expansion of Artificial Intelligence (AI), use in educational and social contexts has changed not only the ways in which individuals search for information, solve tasks, and make decisions, but also the conditions under which they evaluate their own cognitive performance. In particular, generative AI (GenAI) systems and Large Language Models (LLMs) have made access to fluent answers, instant feedback, and conversational support a regular component of everyday cognitive activity. Within this context, the problem of self-evaluation acquires distinct theoretical and practical relevance. It is no longer sufficient to ask whether AI improves performance; it is also necessary to examine whether it supports or, conversely, distorts the individual’s capacity to judge what they understand, control, and can accomplish independently.

In this article, self-evaluation refers to the user’s judgment about their own cognitive performance in relation to a task. Calibration refers to the degree of correspondence between this judgment and actual performance, whereas miscalibration refers to overestimation, underestimation, or the erroneous attribution of success. This distinction is especially important in AI-assisted cognition, because the final product may be improved by the system without a proportional improvement in the user’s own understanding, monitoring, or independent competence. AI-assisted cognition is therefore understood here not as the mere use of a technological tool, but as a task configuration in which AI participates meaningfully in orienting, generating, revising, verifying, or evaluating cognitive activity.

The specific gap addressed in this article is that existing research often examines AI-assisted performance, feedback, self-regulated learning, metacognitive monitoring, or trust in AI as separate issues, without sufficiently explaining how AI-supported task outcomes become cues for users’ judgments of their own competence. This gap is especially relevant in the case of generative AI and large language models, because these systems can produce fluent and apparently high-quality outputs that may improve performance while leaving users uncertain about what they actually understand, control, or can reproduce independently. The article therefore focuses on the conditions under which generative AI, particularly LLMs-based assistance, supports calibrated self-evaluation and the conditions under which it contributes to miscalibration, overestimation, or an illusion of competence.

The theoretical starting point of the article lies in established models of metacognition and self-regulated learning, especially Nelson and Narens’ model of monitoring and control (T. O. Nelson & Narens, 1990) and Winne and Hadwin’s model of self-regulated learning (Winne & Hadwin, 1998). These models are relevant because self-evaluation is not an isolated judgment made after task completion, but part of a broader regulatory process in which users monitor their activity, interpret criteria, compare outcomes with goals, and adjust strategies. In AI-assisted cognition, these processes may be supported, redistributed, or partially externalized to the system. The present framework therefore does not replace established theories of metacognition or self-regulated learning, but reorganizes them under the specific conditions of human–AI interaction.

Achuthan (2025) reports positive effects of AI on self-regulated and self-directed learning, while Pacheco et al. (2025) show that AI-powered learning analytics may support metacognitive and socioemotional competencies when they provide cues for monitoring and adjustment. Tankelevitch et al. (2024) further emphasize that generative AI introduces additional metacognitive demands, because users must formulate prompts, evaluate responses, and decide whether to accept, modify, or reject generated outputs. In the present framework, these findings support the idea that AI can contribute to calibration only when it sustains active monitoring and reflective control. Broader reviews of AI in education and higher education further suggest that these systems should be understood as increasingly stable components of learning ecologies rather than as merely punctual tools. Garzón et al. (2025) and Lai (2024) emphasize implications for autonomy and self-regulation, while Mosha et al. (2026), Navas Bonilla et al. (2025), Vieriu and Petrea (2025), and F. Wu et al. (2025) point to broader effects on academic development and learning environments.

At the same time, a complementary line of research shows that AI may improve the final product without supporting, to the same extent, self-evaluation accuracy, independent understanding, or reflective control over the outcome. Studies on feedback generated by large language models and on performance in reasoning tasks indicate recurrent risks of overestimation and reduced metacognitive accuracy, even when observable performance increases (Fernandes et al., 2026; Liebenow et al., 2025). These findings point to a central tension: AI may make users perform better while leaving them less able to accurately judge the sources, limits, and meaning of that performance.

This tension becomes even more significant when considered in relation to the distinction between performance gains and actual learning progress. Yan et al. (2025) warn that generative AI may produce performance gains without necessarily promoting the deep cognitive and metacognitive processing required for high-quality learning, while Organisation for Economic Co-operation and Development (OECD, 2026) describes a related risk in which apparently improved performance may be mistaken for authentic competence development. AI-assisted products may appear coherent, accurate, or well structured, but these qualities do not necessarily indicate that the user has developed a corresponding level of understanding, transfer capacity, or independent competence. The learning process therefore becomes central for self-evaluation: users may accurately judge their performance only when they can relate the final product to the reasoning, criteria, revisions, and decisions through which it was produced. AI literacy is relevant in this context not as a separate issue, but as a condition that may influence whether users understand the role of the system, recognize the limits of AI-generated output, and distinguish their own contribution from the system’s contribution (Lee et al., 2025; A. S. Nelson et al., 2025; Shi et al., 2025). In such situations, the quality of the AI-assisted product may become a misleading cue for self-evaluation, especially when users cannot independently explain, reproduce, transfer, or justify the result.

Taken together, these findings do not indicate an incoherent literature, but rather a literature that operates at different levels of analysis and captures different configurations of AI use. AI may support self-evaluation when it functions as a scaffold for reflection, criteria clarification, feedback interpretation, and revision. It may contribute to miscalibration when it functions mainly as a mechanism for rapid output production, cognitive substitution, effort reduction, or unverified epistemic authority. The central problem is therefore not whether AI is beneficial or harmful in general, but under what conditions it supports or distorts the user’s judgment about their own performance.

To address this problem, the article organizes the literature around eight analytical axes arranged according to their explanatory sequence: the functional position of AI within the task, the relationship between reflective support and cognitive substitution, metacognitive engagement, effort redistribution, the depth of cognitive engagement, the distinction between assisted performance and actual learning, trust regulation and attribution of success, and self-evaluation accuracy. This ordering reflects the logic of the framework: AI first enters the task in a specific functional position, then operates either as reflective support or as cognitive substitution, thereby influencing the user’s metacognitive engagement, effort distribution, cognitive engagement, the relation between performance and learning, trust regulation, and finally the accuracy of self-evaluation. These axes are closely related, but they do not describe the same construct. In particular, Axis 1 concerns where and when AI enters the task; Axis 2 concerns the cognitive role AI plays as reflective support or cognitive substitution; and Axis 4 concerns how the time and resources saved through AI are redistributed or not redistributed into further cognitive activity.

The framework proposed in this article does not claim that these axes are empirically validated variables in their present form. Nor does it present them as regression models or statistically estimable equations. Instead, the article formulates them as qualitative relational expressions that clarify the direction of the theoretical relationships among the main dimensions of AI-assisted self-evaluation. These expressions are intended to make the explanatory structure of the framework more explicit and to prepare future empirical operationalization.

The article has three aims. First, it clarifies the conceptual relationship between self-evaluation, calibration, metacognitive monitoring, self-regulated learning, and AI-assisted cognition. Second, it positions the proposed framework in relation to foundational models of metacognition and self-regulated learning, especially Nelson and Narens’ model of monitoring and control and Winne and Hadwin’s model of self-regulated learning. Third, it proposes an explanatory framework for understanding the conditions under which AI use supports calibration and those under which it contributes to miscalibration, overestimation, erroneous attribution of success, and the illusion of competence. The broader implication is that AI use should be evaluated not only in terms of performance gains, but also in terms of learning progress, reflective control, and the user’s capacity to form an accurate judgment about their own contribution.

## 2. Methodological Approach

This article follows an integrative conceptual review approach rather than a systematic review or meta-analysis. Accordingly, it does not aim to estimate an overall effect size or to provide an exhaustive mapping of all studies on AI-assisted learning. Its purpose is to identify, compare, and conceptually integrate strands of literature that are theoretically relevant to the problem of calibration and miscalibration in AI-assisted self-evaluation.

The literature was selected through an iterative and purposive process guided by the central concepts of the article: self-evaluation, metacognitive monitoring, judgment calibration, self-regulated learning, cognitive offloading, generative AI, large language models, AI feedback, trust in AI, and AI-assisted learning. Priority was given to studies and reviews that addressed at least one of the following issues: the accuracy of self-assessment, the relationship between performance and learning, the role of AI in metacognitive or self-regulated processes, the delegation of cognitive effort to external tools, and the regulation of trust in automated or AI-based systems.

The review included theoretical articles, empirical studies, systematic reviews, meta-analyses, and conceptual contributions from educational psychology, learning sciences, human–computer interaction, and AI in education. Sources were included when they contributed directly to the construction of the explanatory framework or clarified one of its central dimensions. Sources were excluded when they addressed AI use only in general terms, without a clear connection to self-evaluation, calibration, metacognition, learning processes, cognitive engagement, or trust regulation.

The synthesis was conducted in three steps. First, the literature was examined in order to identify recurring tensions in AI-assisted cognition, especially the distinction between improved task performance and actual learning progress. Second, these tensions were related to established concepts from metacognition and self-regulated learning. Third, the resulting conceptual distinctions were organized into eight analytical axes. The aim of this procedure was to produce an explanatory framework that can guide future empirical research rather than to provide a systematic evidence grading of the literature.

## 3. Theoretical Grounding: Metacognitive Monitoring, Control, and Self-Regulated Learning

The proposed framework is grounded in classical models of metacognition and self-regulated learning. Nelson and Narens’ model of metacognition distinguishes between an object level, where cognitive activity takes place, and a meta-level, where that activity is monitored and controlled (T. O. Nelson & Narens, 1990). Monitoring provides information about the state of cognition, whereas control uses this information to regulate subsequent activity. This distinction is central for the present article because AI-assisted cognition may alter both components. The use of AI may enhance monitoring and revision by offering additional cues, while simultaneously increasing the likelihood that users delegate aspects of monitoring and control to the system.

A second theoretical foundation is provided by Winne and Hadwin’s model of self-regulated learning, in which learners define the task, set goals, enact strategies, monitor progress, and adapt their activity on the basis of feedback (Winne & Hadwin, 1998). From this perspective, self-evaluation is not an isolated judgment made after task completion, but part of a broader regulatory cycle in which learners interpret criteria, compare outcomes with goals, and adjust strategies. AI can enter this cycle in different ways: as a source of feedback, as a scaffold for reflection, as a tool for strategy revision, or as a substitute for cognitive work that would otherwise support learning and self-regulation.

Positioning the present framework in relation to these foundational models clarifies its contribution. The eight axes proposed in this article are not intended as entirely new constructs that replace established theories of metacognition or self-regulated learning. Rather, they reorganize established constructs under the specific conditions of AI-assisted cognition. Their purpose is to explain how the relation between monitoring, control, performance, learning, trust, and self-evaluation changes when part of the cognitive activity is supported, shaped, or generated by AI systems.

## 4. Conceptual Clarifications for AI-Assisted Self-Evaluation

Rather than providing an exhaustive definition of already established terms, this section aims to specify the distinctions without which the analysis of artificial intelligence effects on self-evaluation risks remaining insufficiently differentiated. In its current form, the literature no longer operates convincingly with simple oppositions such as “AI supports” versus “AI distorts” cognitive activity, but rather with finer tensions situated at the intersection of performance, reflection, learning, trust, and the distribution of cognitive control. The framework proposed in this article therefore does not function as a simple taxonomy, but as an analytical instrument through which empirical findings that might otherwise appear contradictory become intelligible. These conceptual distinctions prepare the qualitative relational expressions presented later in the article, without implying that the model has already been empirically estimated.

This need for conceptual differentiation is consistent with the broader literature on metacognition and self-regulated learning, which has consistently shown that monitoring, control, and self-evaluation are not synonymous processes, even though they are closely related. At the same time, more recent reviews of research on self-regulated learning and metacognition suggest that current developments require a recontextualization of these distinctions in learning environments that are increasingly mediated by technology (Dinsmore et al., 2008; Panadero, 2017; Tauber & Ariel, 2023; Yang & Luo, 2025).

A first necessary distinction concerns the relationship between the user’s metacognitive involvement in their own cognitive process and the tendency to externalize to AI precisely those functions that should support self-regulation and monitoring. In the sense adopted here, metacognitive engagement in the process involves the user’s active participation in goal formulation, progress evaluation, response verification, and strategy adjustment. It is not reducible to the mere presence of a technology within the task; rather, it designates the maintenance of a reflective stance toward one’s own cognitive activity. By contrast, avoidance of metacognitive effort designates the tendency to transfer to the system functions that, in a deep learning regime, should remain under the user’s control: the selection of criteria, error detection, comparison of alternatives, or estimation of the adequacy of one’s own response. In AI-assisted contexts, this opposition becomes particularly relevant because generative systems can amplify both the demands of monitoring and the temptation to externalize them (Fan et al., 2025; OECD, 2026; Tankelevitch et al., 2024).

A second distinction concerns the relationship between a simple self-judgment and the adequacy of that judgment in relation to actual performance. In the present article, the adequate evaluation of one’s own performance refers to the correspondence between subjective appraisal and an external or independent performance criterion. It should not be confused with simple self-appraisal, since the latter may also exist under conditions of overestimation, underestimation, or erroneous interpretation of one’s own outcomes. This is precisely why recent literature favors terms such as self-assessment accuracy, performance judgment accuracy, or metacognitive calibration, which capture the relational and measurable character of this correspondence. In AI-assisted contexts, this distinction becomes central because performance may be modified by the system without self-assessment accuracy improving proportionally (Fernandes et al., 2026; Liebenow et al., 2025). In this article, “the adequacy of self-evaluation” designates the operational term, while “self-evaluative calibration” and “self-evaluative miscalibration” designate the broader conceptual framework of the relationship between subjective appraisal and actual performance.

From the perspective of the classical literature on calibration, the issue is not the mere existence of a self-judgment, but the cues users rely on to formulate it and the degree to which these cues correspond to actual performance. Research on calibration, judgments of learning, and self-assessment training has long shown that the accuracy of self-appraisal can be improved when users are oriented toward criteria, explicit monitoring, and reflective task selection; in AI-assisted contexts, this observation becomes even more relevant (Koriat, 1997; Kostons et al., 2012; Lin & Zabrucky, 1998; Stone, 2000; Waldeyer et al., 2024; M. Wu & Was, 2025).

By “AI-assisted cognition,” this article refers not to the mere use of a technological tool, but to a functional configuration in which AI participates meaningfully in orienting, verifying, filtering, reformulating, or generating cognitive activity. The relevant distinction is therefore not between situations “with AI” and “without AI,” but between different ways of integrating technology into the economy of the task. Recent literature suggests that the formative value of AI depends precisely on this functional position: AI may enter the task as support for planning, monitoring, and reflection or, conversely, as a mechanism for the rapid production of an output (Agustin, 2026; Pacheco et al., 2025; Tankelevitch et al., 2024).

From this follows the opposition between AI as reflective support and AI as a substitute for cognitive effort. In the first case, the system makes criteria more visible, provides interpretable feedback, and keeps the user in an active relationship with their own errors; in the second, it provides formulations or solutions that are adopted with minimal verification. For the analysis of self-evaluation, the key issue is not the mere presence of AI, but the effective function it occupies in relation to the user’s cognitive activity (Achuthan, 2025; Agustin, 2026; Fernandes et al., 2026; Liebenow et al., 2025; Pacheco et al., 2025).

At the same time, the dynamics of cognitive delegation cannot be adequately understood without introducing the pragmatic and motivational dimension of time and effort saving. Part of GenAI’s appeal lies not only in its technical capabilities, but also in the promise of reducing the subjective cost of problem solving: less time invested, less effort in formulation, and less cognitive load in the phases of searching, organizing, or drafting. In itself, effort saving is not necessarily problematic; in many contexts, it may free resources for higher-order cognitive operations. The problem arises when time and effort saving becomes the dominant criterion of AI use and shifts the emphasis from understanding and verification to the rapid production of an acceptable result (Lee et al., 2025; OECD, 2026).

This discussion can also be connected to the literature on cognitive offloading, which shows that the externalization of cognitive operations is not in itself dysfunctional, but becomes problematic when it reduces monitoring of one’s own process and modifies the cues on the basis of which users assess their competence. Therefore, the relevant question is not whether the user delegates, but what exactly is delegated, under what conditions, and with what effects on metacognitive control (Dunn & Risko, 2016; Guo & Ye, 2026; Ngai & Gilbert, 2026; Risko & Dunn, 2015; Risko & Gilbert, 2016).

The same observation is directly connected to the distinction between learning progress and performance gains. Yan et al. (2025) warn that GenAI can produce performance gains without promoting the deep cognitive and metacognitive processing required for high-quality learning. OECD (2026) develops this idea as a tension between apparently improved performance and the authentic development of competence. In the present article, performance gain refers to improvement in the observable task outcome, whereas learning progress concerns the consolidation, transfer, and stabilization of competencies that enable the independent and justified completion of similar tasks in the future. Although the two may coexist, they are not equivalent. This distinction is crucial for self-evaluation: if performance is amplified by AI without a proportional increase in learning, the user may come to evaluate their competence on the basis of an output that they cannot independently reproduce, transfer, or justify. This distinction provides the conceptual basis for Axis 6 of the proposed framework, which examines the possible gap between AI-assisted performance and actual learning. It also connects directly to Axis 8, because the accuracy of self-evaluation depends on whether users judge their competence on the basis of independently internalized understanding or on the basis of an AI-assisted product whose production process they cannot fully explain or reproduce.

At this point, the problem of self-evaluation inevitably opens onto the contemporary risks of AI use. One of these concerns the relationship between deep cognitive engagement and reduced cognitive involvement. Deep cognitive engagement involves analysis, elaboration, conceptual integration, critical verification, and the willingness to sustain the difficult processing required for understanding. By contrast, reduced cognitive involvement refers to weaker participation in these processes, usually under conditions of automation, time pressure, or high trust in the generated output. In AI-assisted contexts, this opposition gains additional relevance because fluent responses and the reduction in perceived difficulty may encourage more superficial processing and weaker verification (Lee et al., 2025; OECD, 2026).

The final necessary distinction concerns the relationship between self-judgment and the way users regulate their trust in the external system. In AI-assisted contexts, self-evaluation no longer concerns only the individual’s relationship to their own performance, but also their relationship to the epistemic authority of AI. Kidd and Birhane (2023) show that artificial systems may acquire epistemic authority in ways that shape users’ judgments and dependence, while Wischnewski et al. (2023) emphasize that trust in automated or AI-based systems must be understood in relation to verification, uncertainty, and user control. More recent work on GenAI further suggests that users differ in their willingness to confirm, compare, or suspend acceptance of generated responses (Đerić et al., 2025; Martín-Moncunill & Alonso Martínez, 2025). In the present framework, trust is therefore not analyzed merely as a general attitude toward AI, but as a situated practice of accepting, verifying, or questioning the response. When the system’s contribution becomes opaque and the fluency of the response reduces the impulse toward independent verification, self-evaluation becomes more vulnerable to overestimation and to the erroneous attribution of success.

Therefore, the distinctions proposed here do not aim to establish a definitive taxonomy, but to configure an analytical framework sufficiently fine-grained to make intelligible the divergent findings reported in recent literature. If some studies show that AI can support reflection, feedback, and self-regulation, others indicate that the same technology can amplify overestimation, reduce cognitive involvement, encourage the avoidance of metacognitive effort, or produce an opaque distribution of cognitive control. The point, then, is no longer to define the terms as such, but to explain the conditions under which these dimensions combine in the direction of more adequate self-evaluation and those under which they combine in the direction of miscalibration. Against this background, the next section examines the paradox of self-evaluation in AI-assisted cognition, tracing the tension between its potentially formative and potentially distorting effects.

## 5. Literature Synthesis: The Paradox of Self-Evaluation in AI-Assisted Cognition

This section functions as a synthesis of the reviewed literature. Its purpose is to bring together findings that may appear contradictory when considered separately: studies showing that AI can support reflection, monitoring, and self-regulation, and studies showing that AI can also foster overestimation, reduced cognitive engagement, and miscalibration. The notion of a “paradox” is used here to organize this tension and to prepare the explanatory framework developed in the following section.

After clarifying the relevant conceptual distinctions, the problem can be analyzed as both an empirical and theoretical issue. The reviewed literature does not converge toward a single conclusion. Rather, it suggests that the same AI systems may support reflection, feedback, and monitoring in some configurations, while amplifying overestimation, misplaced confidence, and discrepancies between actual performance and self-appraisal in others. The paradox of self-evaluation should therefore be understood not as a contradiction among studies, but as an indication that current research captures different configurations of the relationship among user, task, and AI system.

In its positive register, recent literature describes AI as an infrastructure of support for reflection, monitoring, and self-regulation. Achuthan’s (2025) meta-analysis indicates overall positive effects on self-regulated and self-directed learning, while the systematic review by Pacheco et al. (2025) shows that AI-powered learning analytics systems can contribute to the development of metacognitive and socioemotional competencies when they provide useful cues for monitoring and adjustment. Tankelevitch et al. (2024) also emphasize that GenAI introduces additional metacognitive demands, because users must formulate prompts, evaluate responses, and decide the extent to which they accept, modify, or reject them. Agustin (2026) develops this line theoretically and proposes understanding AI as an instance of co-regulation in self-regulated learning, while Zhu (2026) shows that the value of these systems depends on how they are integrated into processes of feedback, reflection, and strategic regulation. In this configuration, AI can become a functional support for cognitive activity and can contribute to maintaining an active relationship between the user and their own learning process and performance. This line is complemented by studies on GenAI-mediated self-regulation and critical thinking, which suggest that the formative value of AI depends on the type of activity it sustains within the task (Amzalag, 2026; Lai, 2024; Shi et al., 2025). Other work on feedback, problem solving, and generative chatbot interaction similarly indicates that AI becomes educationally meaningful when it supports examination, revision, and re-elaboration rather than simple answer adoption (Sun et al., 2025; Tsakeni et al., 2025; Wang et al., 2023; Zhou et al., 2024; Zhu, 2026).

However, recent literature also shows increasingly clearly that the mere availability of AI support guarantees neither reflective engagement nor a more adequate appraisal of one’s own performance. Henderson et al. (2025) and Weidlich et al. (2025) show that students appreciate AI feedback for its speed and accessibility, but continue to regard human feedback as more contextualized and more useful for understanding. Jansen et al. (2025) go further, showing that the availability of GPT-4 feedback does not automatically lead to revision and integration. These results suggest that between the existence of AI support and its effective transformation into reflection, monitoring, and adequate self-evaluation, there is a mediating space that cannot be reduced to the mere presence of feedback. Interaction with AI may create favorable conditions for reflection, but these conditions do not automatically lead users to engage in genuine metacognitive actions.

In its critical register, the literature makes visible an increasingly clear rupture between performance and the appraisal of one’s own performance. Liebenow et al. (2025) show that feedback generated by an LLM does not improve, on average, the extent to which self-evaluation corresponds to actual performance, even though it may help initially less well-calibrated users. Fernandes et al. (2026) go further and show that AI can increase performance in reasoning tasks while users overestimate their results more and assess their own level of success less accurately. This finding is particularly important because it makes visible a decoupling between observable success and reflective access to the conditions of that success. What improves at the level of the outcome does not automatically transfer to the level of self-appraisal. In the same register, Galindo-Domínguez et al. (2026) show that certain self-regulatory behaviors may coexist, paradoxically, with excessive dependence on AI, suggesting that technological support and reflective control do not automatically increase together.

The theoretical importance of this tension becomes even clearer when related to the distinction between performance gains and actual learning progress. Yan et al. (2025) warn that GenAI can produce better results without supporting the level of deep cognitive and metacognitive processing required for high-quality learning. Fernandes et al. (2026) provide a clear illustration: AI improves results in reasoning tasks, but not the corresponding metacognition. OECD (2026) turns this observation into a major problem, formulating the risk of a “mirage of false mastery,” that is, apparently good performance that does not reflect authentic cognitive development. Under such conditions, users may have the impression that they have better mastery of a competence precisely because the system produces a high-quality result, even though that result is not grounded in proportional progress in learning, transfer, and conceptual control. Here the paradox of self-evaluation intersects directly with a broader problem of contemporary cognition: performance becomes increasingly less informative about what the user can reproduce, justify, and sustain independently.

This transformation is amplified by an orientation toward saving time and cognitive effort. Lee et al. (2025) show that users frequently report diminished critical engagement when using GenAI, especially in routine tasks, under time pressure, or when trust in the system is high. Fan et al. (2025) show that this effort saving can take the form of avoiding the metacognitive task, while OECD (2026) warns that GenAI often favors “fast, efficient answers over slow, effortful reasoning.” Convergently, research on AI feedback and self-regulated learning shows that the variability of effects also depends on whether the design of the intervention succeeds in maintaining engagement in concrete actions of processing, verification, and integration. Time and effort saving is therefore not merely a pragmatic circumstance, but a central explanatory mechanism: it can make AI attractive and efficient in the short term, but it can also undermine precisely the conditions of a self-evaluation well anchored in one’s own cognitive activity.

In this sense, recent literature on metacognitive laziness, disengagement, and overreliance should be read not as a series of isolated warnings, but as convergent expressions of the same shift. When the task is reorganized around the rapid production of the outcome, metacognitive engagement in the process tends to decrease, cognitive involvement becomes more superficial, and self-appraisal risks relying on an AI-supported result that is erroneously attributed to one’s own competence. This explains why recent literature can describe AI simultaneously as a support for reflection and as an infrastructure for reducing reflective effort. This is not a pure contradiction, but a matter of different regimes of use and different distributions of effort, control, and trust. In the same direction, recent studies suggest that the effects of GenAI on critical thinking differ significantly depending on the mode of use: passive use oriented toward adopting the answer does not have the same cognitive status as collaborative use, in which the system is integrated into a process of examination and re-elaboration (Nasr et al., 2025; Zhao et al., 2025).

The paradox becomes further complicated when the issue of adequately regulating trust in AI systems is introduced. Lee et al. (2025) argue that metacognitive sensitivity is essential for the judicious use of AI recommendations. Kidd and Birhane (2023) show that artificial systems may acquire epistemic authority in ways that shape users’ dependence, while Wischnewski et al. (2023) emphasize that trust in automated systems must be understood in relation to uncertainty, verification, and user control. Under conditions of excessive trust, this distribution of control may become opaque: it is no longer clear what belongs to the user’s own competence and what belongs to the system’s contribution. In this sense, the paradox of self-evaluation also becomes a paradox of trust: success may increase at the very moment when the user loses part of the control over the conditions that make it possible. From this perspective, the paradox of self-evaluation does not require only a narrative description of the positive and negative effects of AI, but also a schematization of the relationships among the factors involved. This need justifies the transition, in the next section, from a synthesis of the literature to the formulation of an explanatory model based on qualitative relational expressions.

Taken together, these developments show that divergent findings in recent literature are not a sign of incoherent research, but rather of the fact that self-evaluation in AI-assisted cognition is a multilayered phenomenon. It depends simultaneously on the degree of metacognitive engagement, the extent to which the appraisal of one’s own performance corresponds to actual performance, the effective function of AI within the task, the relationship between reflective support and cognitive substitution, the economy of time and effort, the difference between learning progress and performance gains, the depth of cognitive engagement, and the way users calibrate trust and distribute control. Therefore, the next step is not the mere accumulation of empirical findings, but the formulation of an explanatory framework capable of showing how these dimensions interact and under what conditions they converge either toward a more adequate appraisal of one’s own performance or toward self-evaluative inadequacy, a false sense of personal efficiency, and distorted self-perception.

## 6. The Proposed Explanatory Model

The model proposed in this section builds on established concepts from metacognition, self-regulated learning, judgment calibration, cognitive offloading, and trust in AI. Its contribution does not consist in introducing entirely new constructs, but in reorganizing these constructs into an explanatory framework suited to the specific conditions of AI-assisted cognition. In this sense, the eight axes should be understood as an integrative structure that translates established theoretical concerns into the context of human–AI interaction.

The eight axes synthesize different strands of existing literature. Axes 1 and 2 build on studies showing that AI may function either as a scaffold for reflection, feedback, and revision or as a mechanism for rapid output production and cognitive substitution (Achuthan, 2025; Pacheco et al., 2025; Tankelevitch et al., 2024). Axes 3–5 draw on research on metacognitive monitoring, self-regulated learning, cognitive offloading, effort saving, and depth of cognitive engagement, which together clarify how AI use can either preserve or reduce the user’s active role in the task (Dunn & Risko, 2016; Fan et al., 2025; Lee et al., 2025; Risko & Gilbert, 2016). Axis 6 is grounded in studies distinguishing AI-assisted performance gains from actual learning progress (Fernandes et al., 2026; OECD, 2026; Yan et al., 2025). Axes 7 and 8 synthesize research on trust in AI, attribution of success, judgment calibration, and self-assessment accuracy (Kidd & Birhane, 2023; Liebenow et al., 2025; Wischnewski et al., 2023). In this sense, the model does not stand apart from the literature reviewed earlier, but reorganizes it into an explanatory sequence focused on calibration and miscalibration in self-evaluation.

The central claim of the model is that AI does not directly produce either self-evaluative calibration or miscalibration. Instead, it modifies the conditions under which users monitor their own activity, regulate cognitive effort, interpret performance, distinguish their own contribution from the system’s contribution, and attribute success. From this reconfiguration, either more adequate self-evaluation or miscalibrated self-evaluation may result.

The framework is organized according to an explanatory sequence. First, AI enters the task through a specific functional position. Second, it operates either as reflective support or as cognitive substitution. Third, this configuration influences the user’s metacognitive engagement. Fourth, it affects the redistribution of time and effort. Fifth, it shapes the depth of cognitive engagement. Sixth, it affects the relationship between assisted performance and actual learning. Seventh, it influences trust regulation and the attribution of success. Finally, these dimensions jointly affect self-evaluation accuracy and may lead either to calibration or to miscalibration.

The axes should therefore be read as interacting dimensions rather than as separate or additive factors. The functional position of AI shapes its role as reflective support or cognitive substitution, and this configuration subsequently affects metacognitive engagement, effort redistribution, cognitive engagement, learning, trust regulation, attribution of success, and self-evaluation accuracy.

The eight axes are therefore not presented as statistically validated variables or as independent empirical factors. They are conceptual dimensions that identify different moments of the same explanatory process. The relationships among the axes are formulated as qualitative relational expressions rather than as quantitative models. These expressions are not intended to assign numerical weight, magnitude, or predictive value to the relations described. Their function is conceptual: they clarify the direction of the theoretical relationships and indicate how particular configurations of AI use may increase or decrease the likelihood of calibration or miscalibration.

In the expressions below, ↑ indicates an increase, ↓ indicates a decrease, → indicates a qualitative pathway, and ⇔ indicates a conceptual association. These symbols express directionality only. They should be read as conceptual devices for organizing theoretical relationships and for preparing future empirical operationalization.

Although the eight axes describe the internal logic of the explanatory framework, their effects should not be understood independently of the context in which AI use takes place. For this reason, the model also includes a set of contextual factors that may influence how the relations among the axes unfold. Task complexity, user expertise, AI literacy, time pressure, and system transparency can affect whether AI use supports reflection and verification or, conversely, encourages substitution, overreliance, and superficial processing. These factors are not treated as additional axes of the model, but as contextual conditions that may strengthen, weaken, or redirect the relations among AI function, user engagement, trust regulation, and self-evaluation accuracy.

**Figure 1:**
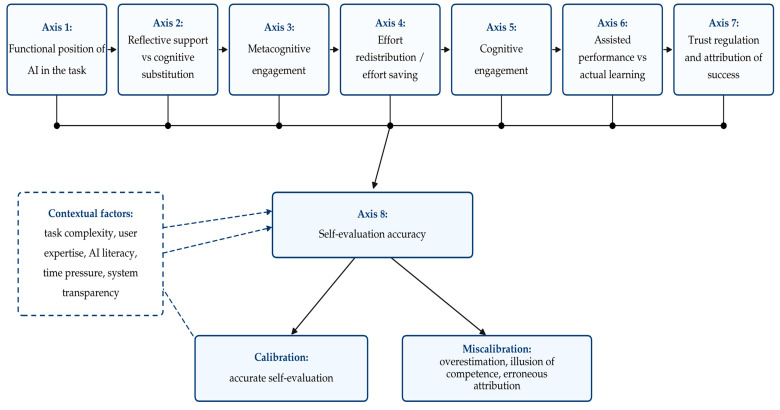
Explanatory framework of self-evaluation in ARTIFICIAL INTELLIGENCE (AI)-assisted cognition.

Figure 1 summarizes the proposed framework and shows how the eight axes interact within a single explanatory sequence.

The figure summarizes the eight analytical axes in their explanatory order. AI first enters the task through a specific functional position (Axis 1) and may operate either as reflective support or as cognitive substitution (Axis 2). This configuration influences metacognitive engagement (Axis 3), effort redistribution (Axis 4), and cognitive engagement (Axis 5), which in turn shape the relation between assisted performance and actual learning (Axis 6) and the regulation of trust and attribution of success (Axis 7). These dimensions jointly affect self-evaluation accuracy (Axis 8), which may result in either calibration or miscalibration. Contextual factors such as task complexity, user expertise, AI literacy, time pressure, and system transparency may influence the strength or direction of these relations. The arrows indicate a conceptual sequence of influence rather than a strictly linear or empirically tested causal chain.

### 6.1. The Functional Position of AI in the Task

The first axis concerns the functional position that AI occupies within the task. Not every use of AI has the same cognitive significance. In some cases, AI is integrated as a support for cognitive activity: it provides cues for task orientation, makes criteria more visible, structures reflection, and facilitates revision. In other cases, it is used mainly as an instrument for the direct production of a result: it delivers an answer, solution, or final formulation without requiring autonomous processing of the content.

This distinction is important because the position of AI within the task shapes the kind of cues available for self-evaluation. When AI supports orientation, clarification, comparison, and revision, users remain more directly connected to the process through which the result is produced. When AI is used primarily to generate a rapid final output, the process becomes less transparent, and the user may evaluate the quality of the product without having sufficient access to the cognitive path that generated it.

This relationship can be expressed as a qualitative relational expression:

AAE ↑ ⇔ Rc ↑, Cv ↑, Sr ↑, Vr ↑, and Of ↓

Rmis ↑ ⇔ Of ↑ and process transparency ↓

In this expression, AAE designates the adequacy of self-evaluation, Rc refers to the provision of cues for task orientation, Cv to the visibility of criteria, Sr to the structuring of reflection, Vr to the facilitation of revision, Of to orientation toward rapid final output, and Rmis to the risk of miscalibration. The expression indicates that self-evaluation is more likely to remain adequate when AI supports the cognitive process rather than merely replacing it with a final product.

Therefore, the model suggests that the effect of AI on self-evaluation depends first on where and how AI enters the task. AI use is more likely to support calibration when it preserves process visibility and gives users cues for judging their own performance. It becomes more likely to contribute to miscalibration when it compresses the process and directs attention mainly toward the final output.

### 6.2. Reflective Support Versus Cognitive Substitution

The second axis concerns the cognitive function performed by AI. AI may function as reflective support when it stimulates comparison, justification, verification, feedback interpretation, and reasoned revision. In this case, the system does not replace the user’s cognitive activity, but helps structure and extend it. By contrast, AI functions as cognitive substitution when it performs central cognitive operations that the user adopts with minimal independent processing.

This distinction clarifies why the same technology can have different effects on self-evaluation. When AI is used as reflective support, the user remains involved in the production and evaluation of the result. When AI becomes a substitute for reasoning, the user may retain the final product but lose access to the reasoning process that would normally support an accurate judgment of competence.

This relationship can be expressed as follows:

AAE ↑ ⇔ RAI ↑ and Vind ↑

Rmis ↑ ⇔ SAI ↑ and Vind ↓

In this expression, AAE designates the adequacy of self-evaluation, RAI designates AI as reflective support, SAI designates AI as cognitive substitution, Vind refers to independent verification by the user, and Rmis refers to the risk of miscalibration. The expression indicates that AI is more likely to support adequate self-evaluation when it stimulates reflection and is accompanied by independent verification. The risk of miscalibration increases when AI substitutes for central cognitive operations and the user performs little or no independent verification.

From this perspective, self-evaluation is influenced not only by the presence of AI, but by the way AI restructures the relation between the user’s own activity and the system’s contribution. The more AI supports comparison, justification, and verification, the more likely the appraisal of one’s own performance is to remain adequate. Conversely, the more AI takes over the core of cognitive activity, and the less the user verifies the generated response, the more vulnerable the judgment of one’s own competence becomes.

### 6.3. Metacognitive Engagement and the Externalization of Monitoring

The third axis concerns the relationship between the user’s metacognitive engagement and the tendency to transfer to AI functions that normally support self-regulation. When users remain involved in formulating goals, selecting criteria, verifying responses, comparing alternatives, and adjusting strategies, AI can function as a support for better-grounded self-evaluation. By contrast, when monitoring is almost entirely externalized and the system assumes the role of a tacit evaluator of the task, users may retain the final product but lose part of their reflective access to the conditions that made that product possible.

This relationship can be expressed as follows:

AAE ↑ ⇔ Mref ↑ and Xmon(1 − Mref) ↓

Rmis ↑ ⇔ Xmon ↑ and Mref ↓

In this qualitative expression, AAE designates the adequacy of self-evaluation, Mref the user’s metacognitive and reflective engagement, Xmon the externalization of monitoring to AI, and Rmis the risk of miscalibration. The expression indicates that self-evaluation is more likely to be adequate when metacognitive engagement is high and monitoring is not delegated to AI without reflective control. Conversely, the risk of miscalibration increases when monitoring is externalized while metacognitive engagement remains low.

The model therefore does not treat externalization as problematic in itself. AI can support self-regulation when users retain control over goals, criteria, verification, and strategy adjustment. It becomes risky when users accept the generated output without sufficiently evaluating the process, the criteria, and their own contribution. In the terms of the model, the adequacy of self-evaluation depends on maintaining reflective involvement in the process, not merely on the quality of the obtained result.

### 6.4. Effort Redistribution and the Reconfiguration of Cognitive Investment

The fourth axis introduces the pragmatic and motivational component of the model. AI use is often associated with the promise of saving time and effort. In itself, this economy is not necessarily problematic; it may be adaptive when it frees resources for higher-level cognitive operations. However, the effect on self-evaluation depends on how the saved resources are redistributed within the task.

If effort reduction becomes the dominant goal of interaction with AI, and the activity is reorganized around the rapid production of a satisfactory result, users may reduce verification, comparison of alternatives, autonomous processing of content, and reasoned justification of the response. In this case, effort saving can weaken the very processes that normally provide cues for accurate self-evaluation.

The risk associated with effort saving can be expressed as follows:

REA ↑ ⇔ EAI ↑ and Iref ↓

In this expression, REA designates the risk of self-evaluative error, EAI the saving of time and effort through AI use, and Iref the reflective reinvestment of saved resources in verification, comparison, elaboration, or conceptual understanding. The expression indicates that effort saving becomes risky for self-evaluation when the resources saved through AI are not reinvested in reflective cognitive activity.

Thus, effort saving has an ambivalent status. It may be beneficial when it reduces repetitive operations and allows the user to invest more in analysis, verification, and conceptual integration. Conversely, it becomes problematic when it is converted into a mere shortening of the cognitive process. In such situations, self-evaluation begins to rely more on the efficiency of interaction with the system than on a sufficiently well-grounded appraisal of the user’s own cognitive contribution.

### 6.5. The Depth of Cognitive Engagement in AI-Assisted Tasks

The fifth axis concerns the depth of cognitive engagement in AI-assisted tasks. Although closely related, metacognitive engagement (Axis 3) and cognitive engagement (Axis 5) are analytically distinct: metacognitive engagement refers to the reflective monitoring and regulation of one’s own cognitive processes, whereas cognitive engagement refers to the depth, persistence, and elaboration with which the task content itself is processed. This dimension does not refer only to the quality of the final product, but to the extent to which the user remains involved in processes of analysis, elaboration, conceptual integration, verification, and critical reformulation of content. In AI-assisted contexts, a correct or fluent result does not necessarily indicate deep cognitive processing; in some situations, it may reflect the system’s capacity to generate a coherent response more than the user’s level of involvement.

Within the proposed model, cognitive engagement functions as a central pathway through which AI use affects self-evaluation. Reflective AI use may sustain or increase cognitive engagement by encouraging users to compare, verify, and revise generated content. Passive AI use may reduce cognitive engagement by encouraging the rapid acceptance of fluent outputs with limited independent processing.

This relationship can be expressed as follows:

Reflective UAI → AC ↑ → AAE ↑

Passive UAI → AC ↓ → Rmis ↑

In this qualitative pathway, UAI refers to the mode of AI use, AC to the depth of cognitive engagement, AAE to the adequacy of self-evaluation, and Rmis to the risk of miscalibration. The expression indicates that reflective AI use may support self-evaluation by maintaining or increasing cognitive engagement. By contrast, passive AI use may weaken self-evaluation by reducing the cognitive engagement that provides users with cues for judging their own performance.

Therefore, the depth of cognitive engagement makes it possible to understand why the same technology can have different effects on self-evaluation depending on the type of activity it supports or replaces. When users analyze, compare, verify, and reformulate AI-generated responses, the appraisal of their own performance is more likely to remain anchored in actual understanding of the task. When AI use reduces perceived difficulty and encourages rapid acceptance of a fluent result, users may evaluate their competence on the basis of a product they have not processed deeply enough.

### 6.6. The Distinction Between Assisted Performance and Actual Learning

The sixth axis concerns the possible gap between improvement in the observable outcome and actual learning progress. In AI-assisted contexts, users may obtain a better product without acquiring, to the same extent, conceptual understanding, argumentative capacity, or the ability to autonomously apply knowledge in similar tasks. This distinction is essential because performance achieved with AI support does not automatically reflect the user’s actual competence.

A correct, coherent, or fluent final product may express the system’s contribution more than the user’s internalized understanding. As a result, the observable outcome can become a potentially misleading cue for self-evaluation, especially when the user cannot independently explain, reconstruct, transfer, or justify the reasoning that led to that result.

The gap between assisted performance and actual learning can be represented as follows:

VAE ↑ ⇔ |PAI − Lactual| ↑

In this expression, VAE designates the vulnerability of self-evaluation to error, PAI the performance or product obtained with AI support, and Lactual the user’s actual learning or internalized competence. The expression indicates that self-evaluation becomes more vulnerable when the gap between AI-assisted performance and actual learning increases.

Conversely, when AI is used in a way that supports elaboration, justification, verification, and application in new contexts, performance and learning remain more closely correlated. In such cases, the appraisal of one’s own performance is more likely to be adequate because the user can connect the final result to internalized understanding and independent competence.

### 6.7. Trust Regulation and the Attribution of Success

The seventh axis concerns the way users regulate trust in AI and attribute success in AI-assisted tasks. The issue is not simply whether users trust AI, but whether this trust is adjusted to the task, to the quality of the response, and to the degree of independent verification. When users can distinguish what belongs to their own contribution from what belongs to the system, AI can be integrated without producing major distortions in self-appraisal. When the system’s contribution becomes opaque and is absorbed into a global representation of success, trust in AI can turn into excessive dependence.

The risk of erroneous attribution of success can be expressed as follows:

RAE ↑ ⇔ TAI > FAI, CAI ↑, OAI ↑, and Vind ↓

In this expression, RAE designates the risk of erroneous attribution of success, TAI the user’s trust in AI, FAI the verified reliability of the AI response, CAI the degree of cognitive control delegated to the system, OAI the opacity of the AI contribution, and Vind the user’s independent verification. The expression indicates that erroneous attribution becomes more likely when trust in AI exceeds verified reliability, cognitive control is delegated to the system, the AI contribution remains opaque, and independent verification is weak.

Final success becomes vulnerable to erroneous attribution when users can no longer sufficiently distinguish between what they produced through their own reasoning and what was generated, corrected, or structured by AI. Under these conditions, the result is claimed too quickly as an expression of one’s own competence, and self-evaluation rests on an insufficiently differentiated representation of the contributions involved. Conversely, when trust is calibrated, the system’s contribution remains transparent, and the response is subjected to independent verification, the risk of erroneous attribution decreases.

### 6.8. Self-Evaluation Accuracy and the Risk of Miscalibration

The eighth axis concerns the central evaluative outcome of the framework: the accuracy of self-evaluation. The issue is not the mere existence of a self-judgment, but the extent to which that judgment corresponds to actual performance. Users may formulate confident appraisals of their own success even when these appraisals are not sufficiently well grounded. Therefore, the central problem is the degree of correspondence between subjective judgment and an external or independently verifiable performance criterion.

The adequacy of self-evaluation can be represented through the relationship between judged performance and actual performance:

EAE = |Jperf − Pactual|

AAE ↓ ⇔ EAE ↑

In this expression, EAE designates self-evaluation error, Jperf the user’s judgment of their own performance, and Pactual the actual performance assessed against external or independently verifiable criteria. The adequacy of self-evaluation decreases as the distance between judged performance and actual performance increases. Overestimation occurs when Jperf > Pactual, whereas underestimation occurs when Jperf < Pactual.

In AI-assisted contexts, this distance may increase when users confuse response fluency, the apparent quality of the final product, or the speed of completion with their own competence. Conversely, when the result is accompanied by verification, justification, and a clear delimitation of the user’s own contribution, the appraisal of one’s own performance is more likely to be adequate. In this final axis, self-evaluation is not treated as an immediate effect of success, but as an outcome of how success is interpreted, verified, and attributed.

### 6.9. Integrating the Eight Axes into a Unified Model

The eight axes do not function as isolated variables, but as moments of the same explanatory sequence. In its unified form, the model can be read as follows: AI first enters the task through a particular functional position; this position determines whether the system operates primarily as reflective support or as cognitive substitution; this configuration influences the user’s metacognitive engagement, the redistribution of time and effort, and the depth of cognitive engagement; the resulting mode of use affects the relationship between assisted performance and actual learning; finally, the user regulates trust in the system and attributes the outcome either in a calibrated and transparent manner or in an excessive and opaque manner. These processes jointly shape the accuracy of self-evaluation and may lead either to calibration or to miscalibration.

In summary, Axes 1 and 2 describe the functional configuration of AI use: where AI enters the task and what cognitive role it plays. Axes 3, 4, and 5 describe the user’s regulatory and cognitive engagement: the extent to which the user remains metacognitively involved, how saved effort is redistributed, and how deeply the task is cognitively processed. Axis 6 explains the relationship between AI-assisted performance and actual learning. Axis 7 concerns the regulation of trust and the attribution of success. Axis 8 concerns self-evaluation accuracy as the central evaluative outcome of the framework.

The model therefore clarifies that self-evaluation accuracy does not depend on a single factor, but on the relational configuration among AI function, user engagement, effort redistribution, learning progress, and trust regulation. AI is more likely to support calibration when it is used as reflective support, when the user remains metacognitively and cognitively engaged, when saved effort is reinvested in verification and elaboration, when assisted performance is accompanied by actual learning, and when trust in the system is appropriately regulated. Conversely, AI is more likely to contribute to miscalibration when it is used as cognitive substitution, when effort saving shortens the task without reflective reinvestment, when performance gains exceed learning progress, and when the system’s contribution is absorbed into an insufficiently differentiated self-appraisal.

## 7. Theoretical and Practical Implications

To avoid analytically restating each of the eight axes, the implications of the proposed framework can be formulated in relation to four groups of axes, each bringing together a core set of problems. Such an organization maintains an explicit connection with the explanatory model without turning this section into a repetition of the previous one. At the same time, it allows for a clearer distinction between what is already well supported in recent literature and what constitutes the specific contribution of the present article. Thus, there is already relevant research on the role of GenAI in learning, the potential of AI to support metacognition, the distinction between assisted performance and actual learning progress, and the risks associated with reduced critical engagement and inadequate regulation of trust in AI systems (Achuthan, 2025; Lee et al., 2025; OECD, 2026; Pacheco et al., 2025; Tankelevitch et al., 2024). What the present article adds is the integration of these lines of research into a unified model of AI-assisted cognitive and metacognitive development, based on the eight axes described through qualitative relational expressions in the previous section. These qualitative relational expressions provide an analytical vocabulary for translating the explanatory framework into research hypotheses, operational indicators, and design criteria for AI-assisted tasks. The implications of the revised eight-axis model are synthesized in Table 1.

### 7.1. Implications Regarding AI Functional Configuration

The first set of implications concerns the functional position of AI within the activity and the effects this position has on the relationship between user and task. The discussion is especially relevant for contexts in which AI participates directly in the elaboration of the response, the structuring of the task, or the revision process.

The literature already suggests that the effects of AI depend not only on its presence, but also on the role it occupies in the activity. Some works indicate that AI can support reflection, monitoring, and self-regulation when it provides interpretable feedback and cues for adjustment (Achuthan, 2025; Pacheco et al., 2025; Tankelevitch et al., 2024). Other studies warn that critical involvement tends to decrease when the system is used primarily to provide a rapid, convenient, or plausible response (Lee et al., 2025; OECD, 2026). From the perspective of the proposed model, AI use should therefore be understood as a form of reorganization of cognitive activity, not as the mere addition of an external tool.

From this perspective, recent research on GenAI-assisted self-regulated learning shows that effects depend on the concrete position the system occupies within the task: support for regulation, feedback resource, exploratory environment, or source of solution. This observation reinforces the idea that the design of AI use must be analyzed at the functional level, not merely at the declarative level (Amzalag, 2026; Lai, 2024; Navas Bonilla et al., 2025; Sun et al., 2025; Tsakeni et al., 2025; Zhou et al., 2024).

Here lies the article’s specific contribution: integrating this observation into a model of self-evaluation that reflects how users construct their self-appraisal. When the technology is used for exploration, comparison, clarification, and revision, users are more likely to remain connected to their own process of strategy elaboration. Conversely, when AI is used mainly to close the task rapidly, the emphasis plausibly shifts from process to final product, which may weaken both cognitive control and the evaluation of one’s own contribution.

Several practical implications follow from this argument. If the functional position of AI matters, then it is not enough to specify whether its use is permitted or not; more important is how and when it is introduced into the task. From the model’s perspective, situations appear more appropriate when AI intervenes after an initial stage of independent analysis or production, as a resource for revision, comparison, or clarification, rather than as a substitute for initial elaboration. Similarly, tasks seem useful when they require justification of choices made in relation to AI suggestions, comparison of versions, and personal re-elaboration of the result.

The same logic also has an institutional dimension. Instead of general rules such as “AI is allowed” or “AI is prohibited,” the model suggests that more productive criteria would be differentiated according to the function the technology occupies in the activity: support for reflection, exploratory tool, feedback resource, or mechanism for rapid response production. Such differentiation can help build contexts of use in which AI expands the capacity for understanding and revision, not merely the speed of execution.

This also suggests possible directions for future empirical operationalization. The dimensions discussed could be examined in future research through indicators such as the timing of AI intervention within the task, the dominant type of system use, the degree of personal re-elaboration after feedback, and the user’s capacity to independently justify the final product. Overall, the model suggests that an important condition of formative AI use is the preservation of a core of personal activity that allows users to remain reflective authors of the process, not merely beneficiaries of the result.

### 7.2. Implications Regarding User Engagement and Effort Redistribution

The second set of implications concerns Axes 3–5: metacognitive engagement, effort redistribution, and the depth of cognitive engagement. These dimensions jointly determine whether AI-assisted activity remains anchored in the user’s reflective and cognitive participation or whether it becomes organized primarily around rapid task completion. The central issue is not only whether users evaluate their own performance, but whether they remain sufficiently involved in monitoring, verification, elaboration, and the reinvestment of saved effort.

Available research shows that the use of GenAI systems involves formulating, monitoring, and evaluating generated responses and that, under certain conditions, these systems can support processes of self-regulation and reflection (Achuthan, 2025; Pacheco et al., 2025; Tankelevitch et al., 2024). At the same time, the mere availability of AI feedback does not, in itself, guarantee a more adequate appraisal of one’s own performance (Liebenow et al., 2025). In the terms of the proposed model, it follows that self-evaluation should be understood not as a global reaction to the result, but as an argued practice dependent on explicit criteria, verification, and the user’s ability to relate the product to their own cognitive contribution.

This interpretation also makes intelligible the findings showing that LLM-generated feedback does not, on average, improve the adequacy of self-evaluation (Liebenow et al., 2025). The present framework suggests that when users relate mainly to the final product and less to their own path of elaboration, the appraisal of their own performance may become more vulnerable to overestimation or insufficient differentiation. The novelty of the article therefore does not lie in reaffirming the general role of metacognition, which is already well documented, but in integrating it into a model of self-evaluation in AI-assisted cognition.

This interpretation is also compatible with the literature showing that self-assessment accuracy can be improved through explicit training in monitoring, task selection, and criteria-based judgment. In the context of the present model, such findings support the idea that adequate self-evaluation is not merely the effect of experience, but also of a task structure that requires guided reflection and justification (Kostons et al., 2012; Stone, 2000; Waldeyer et al., 2024; M. Wu & Was, 2025).

Several practical consequences follow from this interpretation. If the adequacy of self-evaluation depends on maintaining an explicit relationship among process, criteria, and outcome, then AI-assisted activities appear better designed when they make the user’s contribution visible. Within this logic, tasks may be useful when they require an initial independent attempt, followed by AI-assisted revision and an explanation of the differences between the two versions. Similarly, the use of explicit rubrics before and after interaction with AI can create more favorable conditions for self-evaluation anchored in actual understanding of the task, rather than in a general impression of success.

An important modification also concerns the structure of self-evaluation instruments. Instead of general questions such as “How well do you think you did?”, more appropriate formulations are those that require the delimitation of the user’s own contribution and the AI contribution: which part of the response can be independently sustained, which elements were autonomously verified, what was modified after consulting the system, and on what basis. In this form, self-evaluation no longer appears merely as a retrospective reporting procedure, but also as a practice that can support the development of metacognitive discernment.

Without turning this section into a methodological proposal, it is worth adding that the dimensions discussed here could be examined in future research through indicators such as response justification, comparison of initial and revised versions, independent explanation of the final product, and explicit reference to evaluation criteria. It follows that when task design maintains users’ access to their own process of elaboration, the chances of better-grounded self-evaluation also increase.

### 7.3. Implications Regarding the Relationship Between Performance, Learning, and Competence

The third series of implications becomes relevant in contexts where users obtain a high-quality product with AI support, and the central question is no longer only how good the result is, but what it proves about the user’s actual competence.

Recent literature shows that GenAI systems can improve observable performance without supporting, to the same extent, the cognitive and metacognitive processes necessary for robust learning (Fernandes et al., 2026; OECD, 2026; Yan et al., 2025). In the same direction, OECD (2026) warns of the risk of a mirage of false mastery, in which the quality of the final product may create the appearance of competence even though its cognitive basis remains fragile. In the terms of the proposed model, it follows that performance, learning, and competence can no longer be treated as automatically convergent dimensions in AI-assisted contexts.

This problem also appears in studies examining academic performance, GenAI-assisted writing, and the relationship among AI literacy, outcomes, and well-being. Taken together, they suggest that improvement in the final product should not automatically be interpreted as evidence of equivalent conceptual mastery (A. S. Nelson et al., 2025; Shi et al., 2025; Vieriu & Petrea, 2025; Zhao et al., 2025).

The article’s contribution lies in integrating this observation into the analysis of self-evaluation. The model suggests that when users take the final product as a direct indicator of their own competence, without distinguishing between what they can sustain independently and what became possible due to AI support, self-appraisal may become more vulnerable to overestimation. From this perspective, the problem is not only that performance and learning may separate, but also that this separation can affect how users represent their own level of mastery of the task. Here lies one of the central theoretical stakes of the model: self-evaluation must be understood in relation not only to the obtained result, but also to the extent to which that result can be explained, transferred, and sustained independently.

This interpretation has direct practical consequences. If AI-assisted performance cannot automatically be treated as an indicator of competence, then evaluation should not rely exclusively on the final product. From the model’s perspective, more appropriate forms of assessment are those that also include process-related elements: justification of the response, explanation of choices, delimitation of the AI contribution, comparison between an initial and a revised version, and the ability to reformulate or independently sustain the final solution. Consequently, transfer tasks appear particularly relevant, as they can indicate whether the user has acquired only a good result in a punctual situation or also a competence that can be mobilized in related contexts.

Another important consequence concerns the construction of assessment rubrics. Instead of privileging exclusively the correctness and coherence of the final product, rubrics can include distinct criteria for justification, explanation, transfer, and independent support of the response. Such differentiation does not invalidate AI-assisted performance, but reduces the risk of automatically confusing it with fully consolidated competence. In this form, assessment becomes more sensitive to the difference between result, process, and degree of competence internalization.

Here, too, a possible operationalization can be suggested without overloading the methodological dimension of the text. The dimensions discussed could be examined in future research through indicators such as the capacity to independently explain the response, performance in transfer tasks, justification of choices made in relation to AI suggestions, and performance stability in the absence of external support. Overall, the proposed model suggests that a condition of adequate assessment in AI-assisted contexts is avoiding the immediate overlap between the obtained product, actual learning, and the user’s competence.

### 7.4. Implications Regarding Trust Regulation and Evaluative Outcomes

The final group of implications concerns situations in which users must not only use an AI-generated response, but also decide the extent to which they can accept, verify, modify, or claim it as an expression of their own competence.

Recent research shows that the effects of GenAI depend not only on the quality of the generated responses, but also on the extent to which users maintain critical engagement and adequately regulate trust in the system. Lee et al. (2025) show that users often report diminished critical engagement in interaction with GenAI, especially under conditions of time pressure or high trust in the system, while OECD (2026) warns of the risk that fast and fluent responses may favor a logic of easy acceptance at the expense of rigorous verification. In more general terms, these studies suggest that the problem of AI use is not only one of efficiency, but also one of distributing cognitive control and epistemic authority.

The article adds a further dimension here: the relationship between cognitive engagement and trust regulation directly influences how users interpret their own success. When cognitive involvement remains sufficiently high and the AI response is subjected to comparison, verification, and reformulation, users appear better positioned to distinguish what belongs to their own contribution from what belongs to the system. When, by contrast, a fluent response is rapidly accepted without sufficient control, it becomes plausible that the obtained success will be attributed too easily to one’s own competence. Therefore, the problem of trust in AI cannot be separated from the problem of self-evaluation.

This interpretation is also supported by research showing that trust in AI cannot be reduced to a general attitude toward the system. Kidd and Birhane (2023) emphasize the epistemic authority that artificial systems may acquire, while Wischnewski et al. (2023) show that trust in automated systems should be understood in relation to uncertainty, verification, and user control. More recent work on GenAI use further suggests that users differ in their willingness to confirm, compare, or suspend acceptance of generated responses (Đerić et al., 2025; Martín-Moncunill & Alonso Martínez, 2025). From the perspective of the present framework, trust regulation is therefore a situated epistemic practice that directly affects attribution of success and self-evaluation accuracy.

The practical consequences that follow are significant. If responsible AI use involves not only efficient interaction, but also discernment regarding the system’s real limits and contributions, then AI-assisted activities appear more appropriate when they include explicit moments of verification. Useful tasks may include those in which users must indicate which elements of the response they consider sufficiently well grounded, which parts require additional confirmation, and on what basis they accept the final conclusion. Similarly, contexts appear relevant in which users are asked to distinguish between what they actually understand, what they can independently justify, and what they consider merely plausible because of the convincing formulation of the response.

Another consequence concerns AI literacy. Within the framework proposed here, AI literacy should not be reduced to the acquisition of technical skills of use, but should also include the capacity to differentially regulate trust in the system. This involves, for example, sensitivity to task type, to the consequences of error, and to the need for independent verification. In this form, responsible AI use does not mean generalized distrust, but the development of a reflective attitude capable of distinguishing between a convincing response and a sufficiently verified response, between the quality of presentation and the solidity of content.

Without turning this section into a methodological proposal, it is worth noting that the dimensions discussed here could be examined in future research through indicators such as the level of independent verification of the response, the capacity to explain why a response was accepted or rejected, the degree of differentiation between the user’s own contribution and the AI contribution, and the stability of the user’s judgment under conditions of variation in the fluency or apparent authority of the generated response. Overall, the proposed model suggests that one important condition of formative AI use is the preservation of a core of cognitive control that allows users not only to use the response, but also to evaluate it without losing reflective autonomy.

Taken together, these implications converge toward the same conclusion: the central issue is not the mere presence of AI in cognitive activity, but the way it reconfigures the relationship among process, outcome, reflection, and attribution. From a theoretical perspective, self-evaluation must be reconsidered as a judgment situated within a distributed cognitive ecology. From a practical perspective, AI use should be designed in such a way as to maintain the transparency of the user’s own contribution, support verification, and reduce the risk of accepting the result without passing it through personal scrutiny and critical interpretation. In this light, the proposed explanatory model offers not only a scheme for interpreting divergent literature, but also a criterion for designing contexts of AI use that support not only performance, but also more adequate self-evaluation.

## 8. Conclusions

The article started from the observation that the relationship between artificial intelligence and self-evaluation cannot be described in unequivocal terms. Recent literature shows that the same systems can support reflection, monitoring, and self-regulation and, at the same time, foster overestimation, the illusion of competence, and the weakening of the link between actual performance and the appraisal users formulate regarding their own success. Instead of treating these findings as simple contradictions, the article proposed interpreting them within a unified explanatory framework capable of showing that the effects of AI on self-evaluation depend on the concrete configuration of use: the functional position of AI within the task, the level of the user’s metacognitive and cognitive engagement, the relationship between performance and learning, and the way trust in the system is regulated.

The main contribution of the article does not lie in introducing concepts entirely absent from recent literature, but in reorganizing them into a coherent model of self-evaluation in AI-assisted cognition. The eight proposed axes made it possible to correlate lines of research that often appear separately: metacognitive monitoring, the functional status of AI, the economy of time and effort, the difference between performance and learning, the depth of cognitive involvement, and the distribution of cognitive control between user and system. In this form, the model offers not only a descriptive scheme, but also a principle of intelligibility for divergent literature: AI tends to support better-grounded self-evaluation when it remains integrated into a reflective, criteria-based, user-controlled work process; conversely, it tends to foster self-evaluative inadequacy when it is used mainly as a cognitive shortcut, as a mechanism for reducing effort, and as an external instance of evaluation that is either unverified or insufficiently verified.

A distinctive element of the contribution is the formulation of qualitative relational expressions for the eight axes. These expressions do not function as empirically estimated equations, but as conceptual devices that clarify the theoretical direction of the relationships among the main dimensions of AI-assisted self-evaluation. Their value lies in making the explanatory structure of the framework more explicit and in preparing future empirical operationalization.

An important theoretical stake of the approach is that self-evaluation can no longer be treated, in AI-assisted contexts, as a simple subjective reaction to the final product. It must be understood as a judgment situated within a distributed cognitive ecology, in which the outcome is co-produced by user and system, and the central issue becomes the delimitation of one’s own contribution, the transparency of the process, and the epistemic status of the formulated appraisal. More precisely, the article suggests that one of the most significant transformations brought about by AI concerns not only performance or learning, but also the conditions under which individuals come to believe that they understand, control, and master what they have produced.

At the practical level, the proposed model supports the need for contexts of use in which AI is integrated in such a way as to maintain the visibility of the process, the justification of choices, the comparison of alternatives, and the delimitation of one’s own contribution. The general implication is that the design of tasks, assessment, and AI literacy cannot be oriented exclusively toward efficiency, speed, or the immediate quality of the result. It must also aim to preserve the conditions under which users can form a sufficiently well-grounded appraisal of their own performance.

Overall, the article’s contribution consists of proposing an explanatory framework through which the apparently divergent effects of AI on self-evaluation can be understood in a unified way, in relation to the concrete conditions of use. From this perspective, the main issue is not the mere presence of AI, but the way it reconfigures the relationship among outcome, learning, reflective control, and self-perception.

## 9. Limitations and Future Research Directions

The framework proposed in this article has the status of an integrative conceptual model accompanied by qualitative relational expressions. Its first limitation is therefore that it does not provide direct empirical verification of the mechanisms it proposes. The expressions are not statistical equations and are not intended to assign numerical weight, magnitude, or predictive value to the relations described. Their value lies in systematizing theoretical relations, clarifying conceptual pathways, and generating testable hypotheses. Future research should therefore operationalize these dimensions empirically and examine the conditions under which different configurations of AI use influence self-evaluation accuracy.

A second limitation concerns the heterogeneity of the contexts in which artificial intelligence is used. Throughout the article, notions such as “metacognitive engagement,” “functional integration of AI,” “performance,” “learning,” and “trust regulation” have been discussed within a framework general enough to allow the formulation of a unified model. However, these dimensions may take different forms depending on the type of task, the field of activity, the user’s level of expertise, and the practical stakes of the outcome. An academic writing task, an individual learning activity, a professional decision, or a routine task does not activate the same cognitive demands and does not involve the same risks of insufficiently adjusted self-evaluation. For this reason, future research should examine in a more differentiated way how the model applies across distinct educational, professional, and decision-making contexts.

A third limitation is related to the still limited standardization of the indicators through which some of the dimensions discussed could be empirically observed. Although the article suggests cues such as response justification, comparison of versions, the capacity to independently explain the final product, or the delimitation of the AI contribution, these dimensions require more rigorous operationalization. In particular, the adequacy of self-evaluation in AI-assisted contexts cannot be reduced to a single measure, but requires the articulation of several indicators: the correspondence between subjective appraisal and actual performance, the capacity for justification, performance stability in the absence of external support, and the degree of competence transfer. In this sense, an important research direction concerns the development of more refined instruments for measuring self-evaluation under conditions of distributed human–AI cognition.

Similarly, the proposed model should be tested in relation to contextual factors that may influence the relations described. These include the user’s level of expertise, AI literacy, task motivation, time pressure, task complexity, and the type of support provided by the system. It is plausible that the same AI functions may have different effects for novices and advanced users, in exploratory tasks and evaluative tasks, in low-stakes contexts and in contexts where errors have significant consequences. For this reason, a robust empirical verification of the model will need to be sensitive to these differences and avoid treating “AI use” as a homogeneous variable.

In particular, future research should examine more closely the role of AI literacy, differences between students and professionals, the particularities of Science, Technology, Engineering, and Mathematics (STEM) domains, and the possibility of explicit metacognitive interventions that optimize cognitive delegation. It would also be useful to develop research paradigms sensitive to monitoring, feedback use, and concrete forms of verification of the generated response (Amzalag, 2026; Guo & Ye, 2026; Ngai & Gilbert, 2026; Shi et al., 2025; Tsakeni et al., 2025; Wang et al., 2023; M. Wu & Was, 2025).

Another important direction concerns the temporality of the effects. Some current studies capture performance and self-evaluation in the short term, within a task or immediately after interaction with the system. Less clear is what happens in the medium and long term: whether repeated AI use consolidates metacognitive discernment or, conversely, fosters stable forms of cognitive delegation; whether users learn to better regulate trust in the system or become accustomed to treating fluent responses as sufficiently credible. At this point, longitudinal studies become essential, because only they can show whether the effects described here are transient or whether they turn into durable patterns of self-relation and cognitive activity.

Finally, a promising research direction concerns translating the model into principles of pedagogical and institutional design. If the relationship between AI and self-evaluation depends on process transparency, delimitation of one’s own contribution, and the maintenance of reflective control, then the question arises of how contexts of use can be designed to support these conditions. Here, task design, the form of feedback, the timing of AI introduction, assessment criteria, and AI literacy practices all become relevant. Future research could comparatively examine which types of interventions better foster adequate self-evaluation and to what extent certain forms of AI integration can simultaneously support performance, learning, and metacognitive control.

Viewed from this perspective, the proposed model should not be understood as a closed formula, but as a theoretical platform for further investigation. Its usefulness depends precisely on its capacity to generate more precise empirical questions and to orient research toward the concrete conditions under which AI contributes to better-grounded self-evaluation or, conversely, to overestimation, a false sense of competence, and distorted self-perception.

## Figures and Tables

**Table 1:** Theoretical and practical implications of the explanatory model in relation to the revised eight-axis structure.

Axis Group	Analytical Stake	Main Risk	Theoretical and Practical Implication	Qualitative Relational Expression
Axes 1–2: AI functional configuration	How AI enters the task and whether it functions as reflective support or cognitive substitution	AI used mainly as a rapid output generator; reduction in process transparency; substitution of central cognitive operations	AI use should be designed according to its function in the task, not merely allowed or prohibited in general. Tasks should specify whether AI is used for orientation, clarification, feedback, comparison, revision, or direct production. Reflective uses should be encouraged through requirements for independent attempts, justification, and revision.	AI as process support ↑ + independent verification ↑ → self-evaluation accuracy ↑; AI as output generator/cognitive substitution ↑ + verification ↓ → miscalibration risk ↑
Axes 3–5: User engagement and effort redistribution	How users maintain metacognitive engagement, redistribute saved effort, and remain cognitively engaged in the task	Externalization of monitoring; effort saving without reflective reinvestment; superficial processing; reduced cognitive involvement	AI-assisted tasks should preserve the user’s active role in goal formulation, criteria selection, monitoring, verification, and elaboration. Time and effort saved through AI should be reinvested in deeper processing, comparison of alternatives, and conceptual understanding.	metacognitive engagement ↑ + reflective reinvestment ↑ + cognitive engagement ↑ → self-evaluation accuracy ↑; externalized monitoring ↑ + effort saving ↑ + reflective reinvestment ↓ + cognitive engagement ↓ → miscalibration risk ↑
Axis 6: Performance–learning relation	Whether AI-assisted performance corresponds to actual learning, transfer, and independent competence	Confusing a high-quality AI-assisted product with authentic learning or internalized competence; illusion of mastery	Assessment should not rely exclusively on the final product. It should also include explanation, transfer, independent justification, comparison between initial and revised versions, and the ability to reproduce or adapt the result without AI support.	gap between AI-assisted performance and actual learning ↑ → illusion of competence ↑ and miscalibration risk ↑; alignment between performance and learning ↑ → self-evaluation accuracy ↑
Axes 7–8: Trust regulation and evaluative outcome	How users regulate trust in AI, attribute success, and form an accurate or inaccurate judgment about their own performance	Excessive trust in AI; opaque contribution of the system; erroneous attribution of success; overestimation or underestimation of one’s own competence	Responsible AI use requires calibrated trust, explicit verification strategies, and a clear delimitation between the user’s contribution and the AI contribution. Self-evaluation instruments should ask users what they can justify independently, what was generated or revised by AI, and which elements were verified.	excessive trust ↑ + delegated control ↑ + AI contribution opacity ↑ + independent verification ↓ → erroneous attribution and miscalibration risk ↑; calibrated trust + transparent contribution + verification → self-evaluation accuracy ↑

The arrows indicate qualitative directions of theoretical relations, not statistically estimated effects. The “+” sign indicates the joint presence or combination of the specified factors, rather than mathematical addition. “Self-evaluation accuracy” refers to the correspondence between the user’s judgment of performance and an external or independently verifiable criterion. “Miscalibration risk” refers to overestimation, underestimation, or erroneous attribution of success in AI-assisted cognition.

## Data Availability

This study is conceptual and does not involve empirical data; therefore, data sharing is not applicable.
